# Dairy Products: A Potential Source of Multidrug-Resistant *Enterococcus faecalis* and *Enterococcus faecium* Strains

**DOI:** 10.3390/foods11244116

**Published:** 2022-12-19

**Authors:** Marlena Gołaś-Prądzyńska, Magdalena Łuszczyńska, Jolanta Grażyna Rola

**Affiliations:** Department of Hygiene of Food of Animal Origin, National Veterinary Research Institute, 24-100 Pulawy, Poland

**Keywords:** *Enterococcus faecium*, *Enterococcus faecalis*, dairy, resistance genes, antimicrobial resistance, virulence genes, multidrug resistant strains, MDR

## Abstract

This study attempts to present the antimicrobial resistance, virulence and resistance genes of *Enterococcus faecalis* and *Enterococcus faecium* isolated from raw goat’s and sheep’s milk and cheese. Strains were identified by PCR. The dominant species was *E. faecalis* (77.8%) and was most often isolated from raw goat’s milk. The percentage of antimicrobial-resistant *E. faecalis* isolates was higher than that of *E. faecium* isolates, the former most frequently resistant to lincomycin (98%), tetracycline (63%) and streptomycin (16%). Fourteen (22.3%) *E. faecalis* and 2 (11.1%) *E. faecium* isolates were identified as multidrug-resistant (MDR). All MDR *E. faecalis* strains also had virulence genes, whereas one of the two *E. faecium* strains had them. The most prevalent virulence genes in *E. faecalis* isolates were *asa1* (69.8%) and *gelE* (57.1%). The most prevalent resistance genes found in both bacterial species were *tet(M)* (43.2%) and *vgaA* (22.2%). Enterococci from dairy products are confirmed to be a potential source of the spread of antimicrobial resistance, MDR strains, and virulence and resistance genes. This study highlights several aspects of the virulence and pathogenicity of *E. faecalis* and *E. faecium* isolated from dairy products—aspects which are indications for their ongoing monitoring.

## 1. Introduction

One of the main genera of the lactic acid bacteria (LAB) group is *Enterococcus*, comprising over 50 gram-positive species. They are found in a variety of environments, such as the human gastrointestinal tract, animals, plants, soil and water [1]. They can also appear in food of animal origin such as raw meat, fermented sausages and cheeses [2]. Their natural properties, resistance to high temperatures, and the ability to survive in adverse environmental conditions (pH or temperatures) give these enterococci the capacity to survive the pasteurization process and multiply in milk even under refrigeration. For this reason, they are found in both raw and pasteurized milk [3]. Traditionally, in the south and north of Europe they are used as starter cultures to make various, mainly artisanal, cheeses from raw and pasteurized milk [1,3]. Enterococci are a standard element of the non-starter LAB used to produce traditional Mediterranean, Western Balkan countries and Brazilian cheeses from raw milk of sheep, goats and cows [1]. Enterococci also still play an important role in the production of fermented dairy products, such as some cheeses, in extension of the shelf life and improvement of their taste, texture, and aroma [4]. According to data provided by various authors, enterococci may occur naturally or as contaminants of milk and dairy products [5,6,7,8,9,10]. Enterococci isolated from cheese make up one-third of all LAB from this matrix, while they constitute up to 20% of all bacteria isolated from milk [1]. Various species of enterococci are isolated from dairy products; however, strains of *Enterococcus faecium* and *Enterococcus faecalis* remain the most important [3]. Therefore, it is important to study the enterococcal strains derived from dairy products, and enrich the presently insufficient data on the occurrence and virulence factors of isolates derived from milk and dairy products of goats and sheep.

Although enterococci were considered to be microorganisms of low pathogenicity, they have become the main pathogenic factors of nosocomial infections because of their virulence factors, resistance genes and the ease of transfer of genes located on plasmids [11]. *E. faecalis* and *E. faecium* are recognized as opportunistic pathogens and are responsible for an increasing percentage of infections. Enterococci have been shown to be the cause of endocarditis and infections of the urinary tract, bloodstream, and wounds and surgical sites [2,12,13]. Two of the most gravely concerning characteristics of enterococcal food strains are their antibiotic resistance and gene transfer mechanisms.

Both clinical and food-origin enterococci have genomes of high plasticity and plasmids with a wide host range, enabling them to colonize different niches, thereby acquiring and spreading both resistance genes and virulence factors [11]. The therapy of infections with *Enterococcus* spp. etiology in veterinary medicine is mainly based on the results of antimicrobial susceptibility tests and selected for the site of infection and the animal species. The main treatment is monotherapy, which is associated with limited therapeutic options in animals [13]. Enterococci exhibit intrinsic and transferable resistance to several clinically relevant antimicrobial substances, and can easily acquire resistance to others. They are naturally resistant to β-lactam antibiotics and all generations of cephalosporins and sulfonamides. They also show lower susceptibility, to aminoglycosides, lincosamides and quinolones. Resistance to ampicillin, tetracyclines, macrolides, aminoglycosides at high level, chloramphenicol, trimethoprim/sulfamethozaxole, quinolones and streptogramins is not intrinsic but acquired, as is resistance to glycopeptides [5,9,11,13]. Improper, uncontrolled use of antibiotics has led to an increase in the number of resistant strains, including multidrug-resistant strains among enterococci. Enterococcal strains resistant to antimicrobials are widespread in food and are found in meat and dairy products as well as in ready-to-eat foods [5].

Another important aspect related to the severity of *Enterococcus* spp. infections are virulence factors. These factors are responsible for colonization, adhesion to the host’s cells, tissue invasion, evasion of immune responses, and damage to host tissues. We can divide them into virulence factors responsible for colonization, which are an aggregation substance (*asa1*), a collagen binding protein (*Ace*), *E. faecalis* endocarditis-specific antigen (*efaA*), and enterococcal surface protein *esp*, and factors affecting host tissues, these being cytolysin (*cyl*), gelatinase (*gelE*), and hyaluronidase (*hyl*). Enterococcal surface protein encodes a cell-wall surface protein and is involved in the evasion of the host immune response. Its role in the formation of biofilm has also been proven. It occurs mainly in strains of *E. faecalis* [11], but has been shown to be related to ampicillin, ciprofloxacin and imipenem resistance in *E. faecium* [14]. Aggregation substance is a surface adhesion protein which shows enhanced adhesion to renal tubular cells and survival in human macrophages. It is mainly found in *E. faecalis* strains; however, *E. faecium* strains isolated from raw milk were also found positive for the *asa1* gene [12,15]. Cytolysin is a bacteriocin-type exotoxin which has bactericidal properties against gram-negative bacteria and is also toxic to erythrocytes, leukocytes and macrophages. It can occur in a variety of *Enterococcus* spp. including *E. faecalis* and *E. faecium*. Gelatinase E is an enzyme able to hydrolyze gelatin, elastin, collagen, hemoglobin and other bioactive compounds. It is isolated from both clinical and food strains, mainly of *E. faecalis* but also from some of *E. faecium* [14]. The study was aimed to determine the prevalence, antibiotic susceptibility profiles, virulence and resistance determinants of *Enterococcus faecalis* and *Enterococcus faecium* obtained from raw goat’s milk, goat cheese, raw sheep’s milk and sheep cheese.

## 2. Materials and Methods

**Sample collection and isolation of *E. faecalis* and *E. faecium***. A total of 311 dairy product samples were collected from ecological farms located in the southern and western in parts of Poland during 2020–2021. Raw milk and goat cheese samples were obtained from 78 farms, where the number of animals ranged from 3 to 827. Raw sheep’s milk and cheese samples were obtained from 25 farms with between 6 and 750 animals. The samples comprised 216 of raw goat’s milk, 54 of goat’s cheese, 34 of raw sheep’s milk and 7 of sheep’s cheese. Samples were delivered to the Department of Hygiene of Food of Animal Origin in the National Veterinary Research Institute in Poland under temperature-controlled conditions and examined on the day of delivery to the laboratory.

Firstly, 9 mL of the medium supplemented with sodium azide and crystal violet (BioMaxima S.A., Lublin, Poland) was inoculated with 1 mL of raw milk and incubated overnight at 37 °C. Cheese samples of 1 g mass were inoculated into 9 mL of the same medium supplemented with the same sodium azide and crystal violet and likewise incubated overnight at 37 °C. Next, samples with positive growth were inoculated and incubated at 37 °C for a further 24–48 h until clear growth was obtained on Slanetz and Bartley medium (Oxoid Ltd., Basingstoke, Hants, UK). Single colonies with a characteristic growth on the Slanetz and Bartley medium (pink or dark red, with a narrow whitish border colonies were suspected as enterococci) were selected for the next stages: DNA isolation and PCR identification. Cultures were stored in Microbank vials (Pro-Lab Diagnostics, Ontario, ON, Canada) at −70 °C until analysis.

**DNA isolation.** After culturing *E. faecium* and *E. faecalis* strains, their DNA was isolated. Briefly, all *E. faecium* and *E. faecalis* strains were cultivated in brain–heart infusion (BHI) broth (Oxoid Ltd., Basingstoke, Hants, UK) at 37 °C. For DNA extraction, a Genomic Mini Kit (A&A Biotechnology, Gdansk, Poland) was used with material from the 24 h cultures, following the producer’s instructions. A lysis buffer with the addition of lysozyme (A&A Biotechnology, Gdansk, Poland) was used for lysis of the bacterial cells. Proteinase K was used to degrade cellular proteins and release genomic DNA from their binding proteins and to eliminate cellular nucleases. Lysate was bound to the membrane on mini columns, and in the next step, purified DNA was eluted with Tris buffer. Pending analysis, samples were kept at −20 °C.

**PCR identification of *Enterococcus faecium* and *Enterococcus faecalis* species.** The EU reference laboratory for antimicrobial resistance (EURL-AR) methodology was adapted with minor changes to prepare the PCR reaction. Identification was based on *ddlE* gene amplification (D-Ala:D-Ala ligases) specific for each of the species [16]. Primer sets were synthesized and obtained from Genomed (Warsaw, Poland). Each PCR amplification was performed in a 25 µL reaction volume containing 5 µL of template DNA, 1.5 µL (10 pmol/reaction volume) of primers and 13.25 µL of distilled water (MP Biomedicals LLC, Solon, OH, USA), 2.5 µL of 10× PCR Extra Buffer, 0.25 µL of 10 mM MIX dNTP, 1.5 µL of 25 mM MgCl_2_ and 1 U of 1 U/µL polymerase (all products from Thermo Fisher Scientific Baltics, Vilnius, Lithuania unless otherwise noted). The reaction proceeded as follows: initial activation was at 94 °C for 5 min, 30 cycles were run of denaturation at 94 °C for 30 s, annealing at temperatures indicated in Supplementary Table S1, and extension at 72 °C for 1 min, and the reaction was completed with one cycle at 72 °C for 10 min. Positive controls were incorporated as *E. faecalis* ATCC 29212 and *E. faecium* BM4147, as was a negative control as deionised water. For the amplification process, a TProfessional Basic Gradient 96 thermocycler (Biometra/Analityk Jena, Jena, Germany) was utilized. The reaction products were analyzed on a 2% agarose gel in 1×Tris/borate/ethylenediaminetetraacetic acid buffer (EURx, Gdańsk, Poland) and visualized under UV light in a GelDoc XR+ System transilluminator (Bio-Rad, Hercules, CA, USA).

**Detection of resistance and virulence genes.** The extracted DNA of all isolated *E. faecalis* and *E. faecium* strains was screened for the presence of selected resistance and virulence genes. These were the genes most commonly involved in resistance to tetracycline, namely *tet(L)*, *tet(M)* [17] and *tet(W)* [18]; to macrolides and streptogramins, which are *ermB* [19], *vgaA* and *vatD* [20]; and to vancomycin—*VanA* [21] and *VanB* [22]. The reactions proceeded as follows: initial activation was at 94 °C for 3 min, 25 cycles were run of denaturation at 94 °C for 60 s, annealing at temperatures indicated in Supplementary Table S1, and extension at 72 °C for 1 min, and the reaction was completed with one cycle at 72 °C for 10 min. The extracted DNA was screened also for the presence of the *esp, asa1*, *gelE,* and *cylA* genes [23] using a PCR with the corresponding primers (Supplementary Table S1). The reaction proceeded as follows: initial activation was at 95 °C for 10 min, 30 cycles were run of denaturation at 94 °C for 60 s, annealing at temperatures indicated in Supplementary Table S1, and extension at 72 °C for 1 min, and the reaction was completed with one cycle at 72 °C for 10 min.

*Enterococcus faecalis* CG 110::Tn916, *E. faecalis* ENT 14.1 (EQAS 2019), *E. faecalis* JH2-2::Tn1545, *E. faecalis* V583, *E. faecium* BM4145, and *E. faecium* BM4147 were used as positive controls for detection of resistance genes. *Enterococcus faecalis* ATCC 29212 was used as a positive control for detection of virulence genes.

**Antimicrobial susceptibility testing.** Susceptibility was tested by broth microdilution using custom Sensititre EU Surveillance Enterococcus EUVENC Antimicrobial Susceptibility Testing (AST) Plates and Sensititre US National Antimicrobial Resistance Monitoring System Gram-Positive CMV3AGPF AST Plates (Trek Diagnostics, East Grinstead, UK). The assayed panel of antimicrobials comprised 18 antimicrobials (Table 1). *Enterococcus faecalis* ATCC 29212 was used as a reference strain. A 0.5 McFarland suspension of the test microorganisms prepared from a 24 h culture on Slanetz and Bartley medium (Oxoid Ltd., Basingstoke, Hants, UK) was used for the determination of antimicrobial susceptibility. Subsequently, 30 μL of the prepared suspension was added to 11 mL of the liquid medium of cation-adjusted Mueller–Hinton broth with Tris/ethylenediaminetetraacetic acid/sucrose (CAMHBT) buffer (Remel Inc., Lenexa, KS, USA). The next step was transferring 50 μL of the prepared bacterial suspension to each well of the microplate and incubating for 18–24 h at 37 ± 1 °C. Readings of minimum inhibitory concentrations (MIC) were made using a Sensititre Vizion Digital MIC Viewing System (Trek Diagnostics Systems, Cleveland, OH, USA).

For the interpretation of the MIC results, the guidelines of the European Committee for Antimicrobial Susceptibility Testing (EUCAST), the EURL-AR [24] and the Clinical & Laboratory Standards Institute (CLSI) document M100 S29 Performance Standard for Antimicrobial Susceptibility Testing, 29th edition [25] were used. Detailed guidelines are presented in Table 1.

## 3. Results

### 3.1. Identification of E. faecium and E. faecalis from Dairy Products

**Figure 1:**
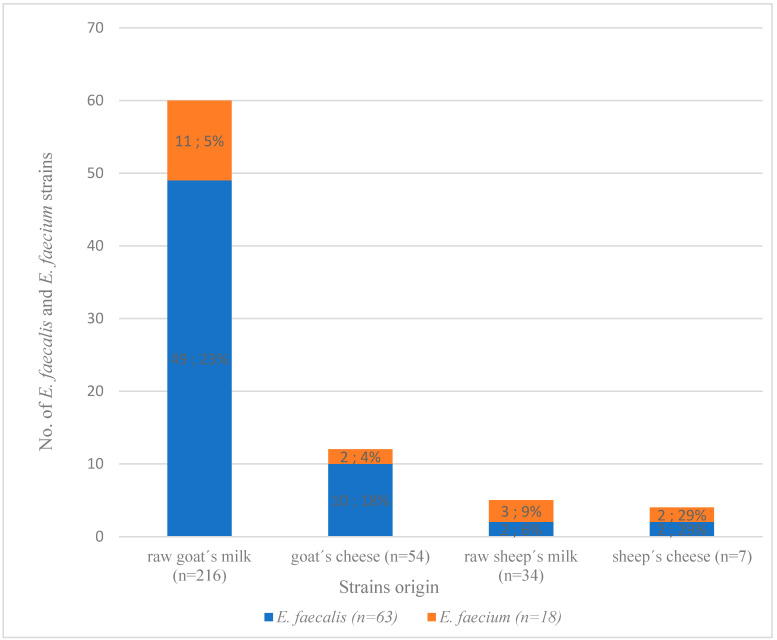
Comparison of the prevalence of *Enterococcus faecium* and *Enterococcus faecalis* isolates in the tested dairy products.

Isolation was successful of 81 (26%) strains of *E. faecium* and *E. faecalis* from the 311 tested samples, and they were 60 (out of 81; 74%) isolates from raw goat’s milk, 12 (out of 81; 15%) from goat’s cheese, 5 (out of 81; 6%) from raw sheep’s milk and 4 (out of 81; 5%) from sheep’s cheese. Each isolate was derived from a single sample. In total, 63 *E. faecalis* and 18 *E. faecium* strains were confirmed by PCR from raw goat’s and sheep’s milk and cheese (Figure 1). Strains of *E. faecalis* were most often isolated from raw goat’s milk (49 out of 63; 78%) and goat’s cheese (10 out of 63; 16%) samples and only single strains were isolated from raw sheep’s milk (2 out of 63; 3%) and sheep’s cheese (2 out of 63; 3%) samples. Strains of *E. faecium* were isolated less frequently from dairy products, most having been taken from raw goat’s milk (11 out of 18; 61%) samples and only single strains originating from the remaining materials. The data are presented in Figure 1.

### 3.2. Antimicrobial Resistance of Enterococcus faecalis and Enterococcus faecium

The results of the determination of antimicrobial resistance using the broth microdilution method for *E. faecalis* and *E. faecium* isolates from dairy products are presented in Table 2. Almost all dairy-derived *E. faecalis* isolates were resistant to quinupristin/dalfopristin (63 out of 63, 100%) and lincomycin (62 out of 63, 98%). In addition, nearly two-thirds were resistant to tetracycline (40 out of 63; 63%). Resistance to streptomycin was indicated by 10 out of 63 (16%), to kanamycin by 9 out of 63 (14%), to tylosin by 7 out of 63 (11%), and to erythromycin by 6 out of 63 (9%). Resistance of *E. faecalis* strains isolated from goat’s milk and cheese to 9 out of the 18 tested antimicrobials was observed, while isolates from sheep’s milk and cheese showed resistance to 3 of the tested antimicrobials. Among the isolates of *E. faecalis* from raw goat’s milk and cheese, the highest resistance rate was shown to tetracycline, respectively, 34 out of 49 (69%) and 6 out of 10 (60%). Interestingly, *E. faecalis* strains isolated from raw sheep’s milk and cheese were only resistant to gentamicin besides quinupristin/dalfopristin and lincomycin.

*Enterococcus faecium* strains isolated from dairy products were resistant to lincomycin in 14 out of 18 isolates (78%), to quinupristin/dalfopristin in 6 out of 18 (33%), to erythromycin in 6 out of 18 (33%), and to gentamicin in 5 out of 18 (28%). Resistance of *E. faecium* strains isolated from raw goat’s milk to 10 out of the 18 tested antimicrobials was observed, while isolates from the remaining materials showed resistance to 2–5 of the tested antimicrobials.

### 3.3. Detection of Virulence and Resistance Genes among Enterococcus faecium and Enterococcus faecalis Isolates

Among all identified *E. faecalis* and *E. faecium* strains, there were no *VanA*, *VanB*, *tet(W)* or *vatD* resistance genes. Only four out of eight resistance genes were detected in the 63 *E. faecalis* strains isolated: *tet(M)*, *vgaA*, *ermB* and *tet(L)*. Only a single *E. faecium* isolate carried antimicrobial resistance genes.

**Figure 2:**
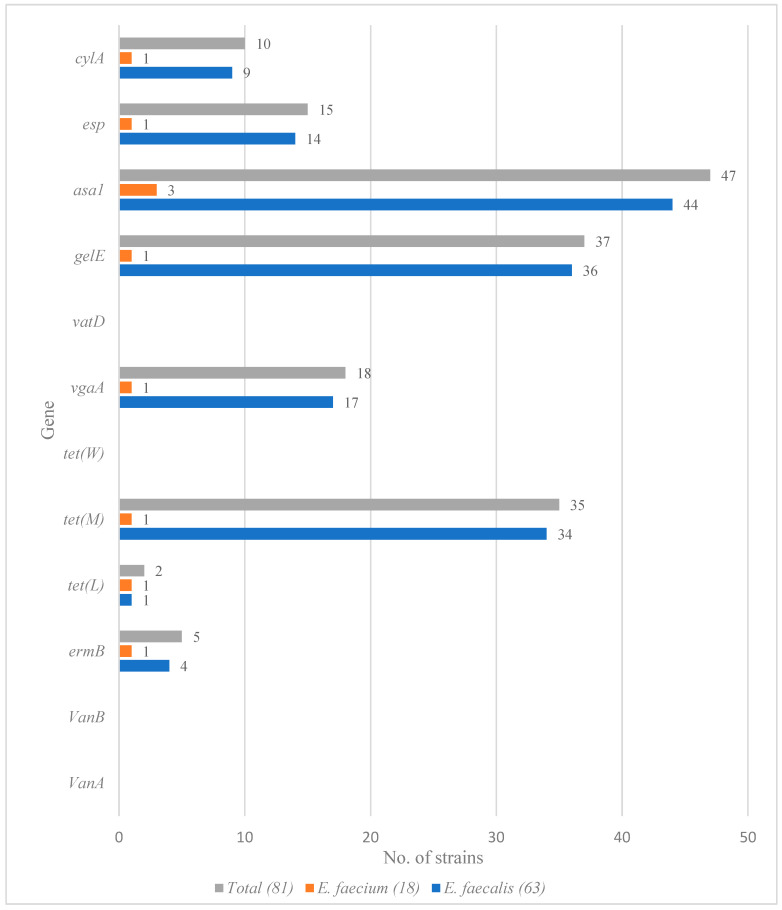
Distribution of virulence and resistance genes among *Enterococcus faecium* and *Enterococcus faecalis*.

The results for the presence of virulence genes were similar. A greater percentage of *E. faecalis* isolates (n = 63) had virulence genes: asa1 was present in 44 out of the 63 strains, *gelE* in 36, *esp* in 14 and *cylA* in 9 strains. Each virulence gene was only possessed by a single *E. faecium* isolate (Figure 2). One *E. faecium* strain harbored all four virulence genes screened for. At least two virulence genes were detected in 28 *E. faecalis* isolates. Four *E. faecalis* strains harbored three virulence genes, and two strains had all four (Table 3).

### 3.4. Connection between Resistance Genes and Phenotypical Resistance to Antimicrobials

The occurrence of resistance genes was compared with phenotypic resistance to antimicrobial agents (Table 4). Out of 40 tetracycline-resistant *E. faecalis* isolates, 33 had the *tet(M)* gene and 1 had the *tet(M)* and *tet(L)* genes. Only one tetracycline-resistant strain of *E. faecium* possessed the *tet(M)* and *tet(L)* genes. Erythromycin-resistant *E. faecalis* strains amount to four (out of the six) possessed the 2-*ermB*, 1-*vgaA,* 1-*ermB* and *vgaA* resistance genes. Of these six erythromycin-resistant *E. faecium* isolates, only one had the *ermB* gene. A 16-strain part of the 63 quinupristin/dalfopristin-resistant *E. faecalis* isolates had the *vgaA* gene, 3 isolates had *ermB*, and 1 had both genes. Of the six resistant strains of *E. faecium*, a single strain harbored the *ermB* gene. Detailed information is presented in Table 4.

### 3.5. Connection between Virulence Genes and Multidrug-Resistant Enterococcus faecalis and Enterococcus faecium Strains

The correlations between virulence gene presence and multidrug-resistant *E. faecalis* and *E. faecium* strains are shown in Table 5. The presence of 14 multidrug-resistant (MDR) strains was found in *E. faecalis* isolates, and those 14 constituted 22.3% of all isolated *E. faecalis*. Isolates resistant to 3 groups of antimicrobial agents (9 out of 63, 14.3%), to 4 groups (3 out of 63, 4.8%) and to 5 groups (2 out of 63, 3.2%) were observed. All multidrug-resistant *E. faecalis* isolates also had virulence genes. Isolates resistant to three groups of antimicrobial agents most often had either the *gelE + esp* genes simultaneously (three strains) or a single *asa1* gene (three strains). Strains of *E. faecalis* resistant to four or five groups of antibiotics simultaneously had at least two virulence genes in different configurations (*gelE + asa1*, *gelE + esp*, or *gelE + asa1 + esp + cylA*). Two multidrug-resistant *E. faecium* isolates were found to be resistant to three groups and six groups of antimicrobial agents, respectively. The strain resistant to six groups of antimicrobial agents also had all the virulence genes screened for.

## 4. Discussion

*Enterococcus* species are frequently occurring bacteria. They are present in various environments from the intestinal tracts of humans and farm animals to diverse forms of food and feed [2]. The most common *Enterococcus* spp. are *E. faecium, E. faecalis,* and *E. durans* which, excluding *E. durans*, were claimed in different studies to be the predominant species in most of the milk and dairy products intended for direct consumption [4,26]. Enterococci are a global health problem and are considered opportunistic pathogens and, in fact, these bacteria have been reported to be associated with some human and animal infections [27]. There are particular challenges in the treatment of enterococcal infections presented by antimicrobial-resistance and virulence genes. These genes may undergo horizontal transfer, which is one of the main mechanisms of their spread among enterococcal species. For this reason, the presence of enterococci in milk may be the cause of strains’ acquisition of MDR [26]. The use of antibiotics in food animals is noted to be associated with rates of antibiotic resistance in humans [11].

Enterococci are often associated with raw milk and cheeses [28,29]. Raw goat’s milk provided 60 of the 81 enterococcal isolates taken from the 311 samples examined, goat’s cheese yielded 12 isolates, sheep’s milk 5, and sheep’s cheese 4. We identified 63 (77.8%) isolates as *E. faecalis* and 18 (22.2%) as *E. faecium. Enterococcus faecalis* as the predominant species in the tested materials. The present study demonstrated that *E. faecalis* was the most common in the tested dairy products, which was in agreement with the findings of other studies [2,9,27,29]. Jahansepas et al. even isolated fivefold more *E. faecalis* (83.33%) than *E. faecium* (16.67%) from cheese samples in Iran [27]. However, one report stated a higher frequency of *E. faecium* than *E. faecalis* in Serbian cheeses [30]. A previous study showed that the occurrence of one *Enterococcus* species relative to another is seasonally dependent: *E. faecalis* was predominant in cold seasons and *E. faecium* in warm seasons [8].

Antibiotic resistance is considered a key factor determining the risk profile of enterococci. The tested *E. faecalis* strains were susceptible to eight antimicrobials: ampicillin, daptomycin, linezolid, teicoplanin, tigecycline, vancomycin, nitrofurantoin, and penicillin. In contrast, it was only to a single drug that *E. faecium* strains did not demonstrate resistance: ciprofloxacin. In the study by Silvetti et al., all tested isolates were susceptible to clinically relevant antibiotics, specifically vancomycin, ampicillin and penicillin G [31]. In Gaglio et al., out of a total of 40 tested strains, the authors observed no resistance to penicillin, ampicillin, vancomycin or linezolid [32]. The same results were obtained for the *E. faecium* and *E. faecalis* isolates tested in this study. Antimicrobial-resistant *E. faecalis* strains comprised a higher percentage of all the strains of that species in this study, than resistant *E. faecium* strains did of all strains of its species. Our observations were in line with previous studies, finding that *E. faecalis* isolates were more resistant to antimicrobials than *E. faecium* isolates [27,33,34]. All *E. faecalis* bacteria isolated were not susceptible to quinupristin/dalfopristin because of their intrinsic resistance to this agent, while among *E. faecium*, six (33%) isolates were resistant to this agent. A higher percentage of *E. faecium* strains resistant to synercid (the trade name of quinupristin/dalfopristin) than in our study was obtained by Hosseini et al., who noted 57.6% not to be susceptible [2]. Regarding tetracycline, we observed 63% of *E. faecalis* isolates to be resistant. This accords with what was reported by Hosseini et al., who analyzed enterococci isolated from meat and dairy products [2]. Similar findings were also presented by other authors [26,29,35]. For both *E. faecium* and *E. faecalis*, as many as 95% of strains were observed to be chloramphenicol-susceptible, an identical outcome to that in the study by Palmieri et al. [29].

Previous investigations have reported varied prevalences of resistant enterococci to erythromycin, which were as follows: 14.3% [36], 46.2% [26], 52.5% [32], 53% [29], and 71.4% [4]. In this study, echoing the results of Chajęcka-Wierzchowska et al. [36], the percentage of erythromycin-resistant enterococci was 14.8%, while in the research of other authors this percentage was higher and ranged from 46.2 to 71.4% [4,26,29,32]. Różańska et al. noted 82.16% of enterococci isolated from mastitis samples to be lincomycin-resistant [37]. Our results showed a larger proportion of enterococci resistant to lincomycin in dairy products.

The presence of multidrug-resistant enterococcal strains is associated with easy spread of resistance genes and virulence genes by horizontal transfer. A total of 16 out of 81 isolates were resistant to at least three antimicrobials, which constituted 19.7% of isolates showing a multidrug-resistant phenotype. In contrast, other authors obtained higher percentages of MDR strains, which were 48.5% [26] and 67.6% [9]. For this reason, consumers need to be aware of the health risks associated with the consumption of raw milk or dairy products, which may be a source of *E. faecalis* and *E. faecium* strains and, in particular, multidrug-resistant phenotypes.

Enterococci are naturally resistant to various antimicrobial agents and have the ability to transfer resistance genes to other microorganisms through plasmid conjugation mechanisms. There was an association between the results of phenotypic antimicrobial resistance tests and the presence of resistance genes among *E. faecalis* strains. As reported by Ruiz et al., complete compliance was noted for tetracycline and erythromycin in most of the resistant strains of tested species, while discrepancies were observed for vancomycin [8]. Almost all tested *E. faecium* and *E. faecalis* strains harbored *tet(L)* and *tet(M)*: 92.1% and 94.7%, respectively. Interestingly, *E. faecium* isolates did not show phenotypic resistance, but did have vancomycin resistance (*VanB*) genes. This may be an example of silent resistance genes as reported by Ruiz et al. [8]. In our study, most tetracycline-resistant strains possessed resistance genes but we did not observe strains phenotypically and genotypically resistant to vancomycin. Similar results were obtained by Różańska et al., demonstrating a very low percentage of vancomycin-resistant *Enterococcus* strains of 0.94% [37]. We reported lower results for *ermB*, while El-Zamkan et al., Ahmed et al., and Jamet et al. detected this gene at a higher frequency [9,38,39]. A similar low incidence of *ermB* occurrence does exist in the literature; comparable incidences of this gene were found by Erbas et al. and Cui et al. [40,41].

In addition to resistance genes, we investigated four virulence determinants: aggregation substance (*asa1*), gelatinase (*gelE*), cytolysin (*cylA*), and enterococcal surface protein (*esp*). The virulence factors of enterococci can contribute to the severity of pathogenesis and disease severity in humans and other animals [26]. Thirty-four (53.9%) of the 63 *E. faecalis* strains possessed at least two virulence genes, and most of them harbored multiple profiles. The very low presence of virulence determinants among *E. faecium* strains in the present study did not disaccord with previous studies, in which their incidence among *E. faecium* strains was notably lower than that in *E. faecalis* strains [6]. Jahansepas et al. reported that *E. faecium* isolates were typically clear of virulence genes, whereas each tested *E. faecalis* strain revealed multiple profiles of such genes [27]. Similar results were reported by Yoon et al. and Yang et al., in whose research *asa1* and *gelE* were the most prevalent genes possessed by *Enterococcus* ssp. strains [26,42]. These results agree with an observation in the work of Jiménez et al. and İspirli et al., who saw that gelatinase was one of the most common virulence factors obtained from enterococcal bacteria from both raw and pasteurized milk [43,44]. Yoon et al. observed a low percentage of the *esp* gene in *E. faecalis* (15.1%) and *E. faecium* (3.0%) strains isolated from milk, which is consistent with our results [26]. Other authors confirmed the role of the *esp* gene in adhesion to host cells and biofilm formation, but also believed that the *gelE* gene may contribute to these processes [9,45]. The incidences of the *cylA* gene in *E. faecalis* and *E. faecium* strains, at 14.3% and 5.5%, respectively, were low in the present investigation. An even lower percentage of this gene in *E. faecalis* strains at 6.6% was obtained by Yoon et al. [26], while Silvetti et al. [31] did not detect this gene at all within their tested strains. The prevalence of virulence factors and the high level of resistance to many different antimicrobials elevate *Enterococcus* spp. and, in particular, *E. faecalis*, to significance as opportunistic agents of nosocomial infections. This is also indicated by the work of another author, in which strains of *E. faecalis* were the most frequently isolated from goat’s milk and characterized by multiple resistance to antimicrobials [46]. Food-borne disease is another problem caused by certain strains of species of this genus, because milk containing multidrug-resistant and virulent *Enterococcus* spp. can be a reservoir for both virulence and antimicrobial resistance genes and the constitution of dairy products with such milk can transfer the genes to the human microflora in the food chain.

## 5. Conclusions

Although enterococci are generally recognized as non-pathogenic microorganisms, strains that are resistant to antibiotics and express various virulence factors have emerged. These factors may be the cause of their high pathogenicity and infectivity. For this reason, enterococci isolated from raw goat’s and sheep’s milk and cheeses should be considered a potential risk to public health. This study presents *E. faecalis* as the most prevalent agent found in raw goat’s and sheep’s milk and cheeses, and illuminates the antimicrobial resistance and virulence profiles of this species and *E. faecium*. A high ratio of antimicrobial resistance resulted, especially to those drugs also applied in human medicine such as tetracycline, lincomycin and streptomycin. We detected 19.7% of isolates to be MDR. All multidrug-resistant *E. faecalis* strains also possessed virulence genes. Compared to *E. faecalis, E. faecium* isolates showed lower resistance to the tested antimicrobial agents and had fewer virulence and resistance genes. Importantly, we did not observe isolates resistant to linezolid and vancomycin, antibiotics important in human medicine. The isolates of greater concern were of *E. faecalis*, because they showed resistance to antimicrobials more consistently and had more virulence and resistance genes.

The results of this study confirmed that enterococcal strains from raw goat’s and sheep’s milk and cheeses might be a potential source of the spread of antimicrobial resistance conferred by MDR strains and virulence and resistance genes. This hazard raises the importance of informing both milk producers and veterinarians about the effects of improper use of antimicrobials and thus reducing the spread of resistant isolates, including multidrug-resistant isolates, to humans through the food chain.

## Figures and Tables

**Table 1:** Breakpoints for *Enterococcus faecalis* and *Enterococcus faecium* according to EURL guidelines adopted from EUCAST [24] and CLSI [25].

Antimicrobial Substance	*E. faecium*	*E. faecalis*
	MIC (μg/mL) R is >	MIC (μg/mL) R is >
Ampicillin, AMP	4	4
Chloramphenicol, CHL	32	32
Ciprofloxacin, CIP	4	4
Daptomycin, DAP	8	4
Erythromycin, ERY	4	4
Gentamicin, GEN	32	32
Linezolid, LZD	4	4
Quinupristin/Dalfopristin (Synercid), SYN	4	Intrinsically resistant
Teicoplanin, TEI	2	2
Tetracycline, TET	4	4
Tigecycline, TGC	0.25	0.5
Vancomycin, VAN	4	4
Kanamycin, KAN	1024 *	1024 *
Lincomycin, LIN	8 *	8 *
Nitrofurantoin, NIT	64	64
Penicillin, PEN	16 *	16 *
Streptomycin, STR	512	512
Tylosin, TYL	32 *	32 *

* breakpoints according to CLSI guidelines.

**Table 2:** Number (%) of antibiotic-resistant *Enterococcus faecium* and *Enterococcus faecalis* strains isolated from goat- and sheep-origin dairy product samples.

Material	*E. faecalis* Isolates (n = 63)					*E. faecium* Isolates (n = 18)				
	Raw Goat’s Milk (49)	Goat’s Cheese (10)	Raw Sheep’s Milk (2)	Sheep’s Cheese (2)	Total (63)	Raw Goat’s Milk (11)	Goat’s Cheese (2)	Raw Sheep’s Milk (3)	Sheep’s Cheese (2)	Total (18)
Antimicrobial substance										
Ampicillin	0	0	0	0	0	0	0	0	0	0
Chloramphenicol	3 (6%)	0	0	0	3 (5%)	1 (9%)	0	0	0	1 (5%)
Ciprofloxacin	1 (2%)	0	0	0	1 (2%)	0	0	0	0	0
Daptomycin	0	0	0	0	0	0	0	0	0	0
Erythromycin	5 (10%)	1 (10%)	0	0	6 (9%)	5 (45%)	1 (50%)	0	0	6 (33%)
Gentamicin	0	0	2 (100%)	1 (50%)	3 (5%)	1 (9%)	0	3 (100%)	1 (50%)	5 (28%)
Linezolid	0	0	0	0	0	0	0	0	0	0
Quinupristin/Dalfopristin	49 (100%)	10 (100%)	2 (100%)	2 (100%)	63 (100%)	4 (36%)	2 (100%)	0	0	6 (33%)
Teicoplanin	0	0	0	0	0	0	0	0	0	0
Tetracycline	34 (69%)	6 (60%)	0	0	40 (63%)	1 (9%)	0	0	0	1 (5%)
Tigecycline	0	0	0	0	0	0	0	0	0	0
Vancomycin	0	0	0	0	0	0	0	0	0	0
Kanamycin	8 (16%)	1 (10%)	0	0	9 (14%)	2 (18%)	0	1 (33%)	0	3 (17%)
Lincomycin	48 (98%)	10 (100%)	2 (100%)	2 (100%)	62 (98%)	8 (73%)	2 (100%)	3 (100%)	1 (50%)	14 (78%)
Nitrofurantoin	0	0	0	0	0	1 (9%)	1 (50%)	0	0	2 (11%)
Penicillin	0	0	0	0	0	0	0	0	0	0
Streptomycin	9 (18%)	1 (10%)	0	0	10 (16%)	1 (9%)	0	0	0	1 (5%)
Tylosin	6 (12%)	1 (10%)	0	0	7 (11%)	1 (9%)	1 (50%)	0	0	2 (11%)

**Table 3:** Multiple virulence genes patterns in *Enterococcus faecalis* and *Enterococcus faecium* isolates.

Virulence Genes	*E. faecalis* (63)	*E. faecium* (18)	Total (81)
*gelE + asa1*	15 (24%)	0	15 (18%)
*gelE + esp*	8 (13%)	0	8 (10%)
*asa1 + cylA*	5 (8%)	0	5 (6%)
*gelE + asa1 + esp*	2 (3%)	0	2 (2%)
*asa1 + esp + cylA*	1 (2%)	0	1 (1%)
*gelE + asa1 + cylA*	1 (2%)	0	1 (1%)
*gelE + asa1 + esp + cylA*	2 (3%)	1 (5%)	3 (4%)

**Table 4:** Resistance gene patterns in *Enterococcus faecalis*- and *Enterococcus faecium*-resistant strains.

Antimicrobial Substance	Profile	*E. faecalis* (n = 63)	*E. faecium* (n = 18)
Tetracycline	R	40	1
	*tet(M)*	33	0
	*tet(L)*	0	0
	*tet(M) + tet(L)*	1	1
Erythromycin	R	6	6
	*ermB*	2	1
	*vgaA*	1	0
	*ermB + vgaA*	1	0
Quinupristin/Dalfopristin	R	63	6
	*ermB*	3	1
	*vgaA*	16	0
	*ermB + vgaA*	1	0

R—strains phenotypically resistant to antimicrobial substances.

**Table 5:** Virulence genes patterns in *Enterococcus faecalis* and *Enterococcus faecium* multidrug-resistant (MDR) strains.

	Number of Antimicrobial Substance Groups	MDR Strain Resistance Profile	Virulence Gene Profile
*E. faecalis* (n = 63)	3	TET + LIN + STR (1 *)	*asa1*
		ERY + TET + LIN (2 *)	*asa1* (2 *)
		TET + KAN + LIN (1 *)	*asa1 + esp + cylA*
		TET + LIN + STR + KAN (5 *)	*gelE + esp* (3 *)
			*gelE* (1 *)
			*esp* (1 *)
	4	TET + LIN + STR + CIP (1 *)	*gelE + asa1 + esp + cylA*
		CHL + ERY + TET + LIN (1 *)	*gelE + asa1*
		ERY + TET + KAN + LIN + STR (1 *)	*gelE + asa1*
	5	CHL + ERY + TET + KAN + LIN + STR (2 *)	*gelE + esp* (2 *)
*E. faecium* (n = 18)	3	ERY + LIN + NIT (1 *)	-
	6	CHL + ERY + GEN + TET + KAN + LIN + STR (1 *)	*gelE + asa1 + esp + cylA*

* No. of multidrug-resistant (MDR) strains, CHL—chloramphenicol, CIP—ciprofloxacin, ERY—erythromycin, GEN—gentamicin, KAN—kanamycin, LIN—lincomycin, NIT—nitrofurantoin, STR—streptomycin, TET—tetracycline.

## Data Availability

The data presented in this study are available upon request from the corresponding author.

## Supplementary Materials

The following supporting information can be downloaded at: https://www.mdpi.com/article/10.3390/foods11244116/s1, Table S1: Oligonucleotide primers used in this work.
