# Supplementary material for: Dairy Products: A Potential Source of Multidrug-Resistant Enterococcus faecalis and Enterococcus faecium Strains
Source: Foods. 2022 Dec 19;11(24):4116. doi: 10.3390/foods11244116 (PMC9778350; doi:10.3390/foods11244116)
Supplement: Supplementary file 1 [file foods-11-04116-s001.zip › Supplementary Materials 2022.11.18 wysêane.pdf]

Supplementary Materials

Table S1. Oligonucleotide primers used in this work.

Genes	Sequence (5′–3′)	Annealing	Amplicon size (bp)	Reference
ddl E. faecalis	F: ATCAAGTACAGTTAGTCTT<br>R: ACGATTCAAAGCTAACTG	50	941	[16]
ddl E. faecium	F: GCAAGGCTTCTTAGAGA<br>R: CATCGTGTAAGCTAACTTC	50	550	
16S	F: AGAGTTTGATCCTGGCTNAG<br>R: GACTACCNGGGTATCTAATCC	50	800	
tet(L)	F: ATTACACTTCCGATTTCGG<br>R: CATTTGGTCTTATTGGTACG	54	1550	[17]
VanA	F: AACAACTTACGCGGCACT<br>R: AAAGTGCGAAAAACCTTGC	55	1550	[21]
ermB	F: GGAACATCTGTGGTATGGCG<br>R: CATTTAACGACGAAACTGGC	52	426	[19]
VanB	F: GAT ATT CAA AGC TCC GCA GC<br>R: GGT ATC TTC CGC ATC CAT CA	55	1000	[22]
tet(M)	F: GTTAAATAGTGTTCTTGGAG<br>R: CTAAGATATGGCTCTAACAA	45	1500	[17]
tet(W)	F: TGG TCC CCT AAT ACA TCG TT<br>R: GCC ATC TTG GTG ATC TCC	55	1550	[18]
vgaA	F: AGT GGT GGT GAA GTA ACA CG<br>R: CTT GTC TCC TCC GCG AAT AC	55	200	[20]
vatD	F: GCT CAA TAG GAC CAG GTG TA<br>R: TCC AGC TAA CAT GTA TGG CG	55	330	
gelE	F: TATGACAATGCTTTTTGGGAT<br>R: AGATGCACCCGAAATAATATA	56	213	[23]
asa1	F: GCACGCTATTACGAACTATGA<br>R: TAAGAAAGAACATCACCACGA	56	375	
esp	F: AGATTTCATCTTTGATTCTTGG<br>R: AATTGATTCTTTAGCATCTGG	56	510	
cylA	F: ACTCGGGGATTGATAGGC<br>R: GCTGCTAAAGCTGCGCTT	56	688	
